# Evaluation of Ruxolitinib for Steroid-Refractory Chronic Graft-vs-Host Disease After Allogeneic Hematopoietic Stem Cell Transplantation

**DOI:** 10.1001/jamanetworkopen.2020.34750

**Published:** 2021-01-27

**Authors:** Hengwei Wu, Jimin Shi, Yi Luo, Yamin Tan, Mingming Zhang, Xiaoyu Lai, Jian Yu, Lizhen Liu, Huarui Fu, He Huang, Yanmin Zhao

**Affiliations:** 1 Bone Marrow Transplantation Center, First Affiliated Hospital, School of Medicine, Zhejiang University, Hangzhou, Zhejiang, People’s Republic of China; 2 Institute of Hematology, Zhejiang University, Hangzhou, Zhejiang, People’s Republic of China

## Abstract

**Importance:**

Ruxolitinib, a selective inhibitor of the Janus kinases 1/2 signaling pathway, has shown a significant response in steroid-refractory chronic graft-vs-host disease (SR-cGVHD), a major cause of morbidity and mortality in individuals who have undergone allogeneic hematopoietic stem cell transplantation (HSCT).

**Objectives:**

To investigate the clinical response to ruxolitinib in patients with SR-cGVHD after allogeneic HSCT and to evaluate its safety profile during the treatment course.

**Design, Setting, and Participants:**

This single-center case series included 41 consecutive patients who were treated with ruxolitinib for SR-cGVHD after allogeneic HSCT between August 2017 and December 2019. Data were collected from each patient’s medical record at the First Affiliated Hospital of Zhejiang University School of Medicine. Data analysis was conducted from March to May 2020.

**Exposure:**

Ruxolitinib.

**Main Outcomes and Measures:**

Treatment responses, factors associated with response, and adverse effects during ruxolitinib administration.

**Findings:**

Overall, 41 patients (median [range] age, 31 [17-56] years; 14 [34.1%] women) were treated with ruxolitinib and included in this study. A total of 15 patients (36.6%) had a complete remission, and 14 (34.1%) had a partial remission, with an overall response rate of 70.7% (29 patients; 95% CI, 56.2%-85.3%). Lung involvement (odds ratio, 0.112; 95% CI, 0.020-0.639; *P* = .01) and matched related donors (odds ratio, 0.149; 95% CI, 0.022-0.981; *P* = .048) were associated with less favorable treatment response. Major adverse events associated with ruxolitinib were cytopenias and infectious complications. The median (range) follow-up for this cohort was 14.9 (1.4-32.5) months. Prolonged survival was observed in patients with a male donor (*P* = .006), complete remission before transplantation (*P* = .02), baseline moderate cGVHD (*P* = .02), and skin cGVHD (*P* = .001).

**Conclusions and Relevance:**

In this small, single-site case series, ruxolitinib demonstrated a significant response in heavily pretreated patients with SR-cGVHD and a reasonably well-tolerated safety profile. The results add to the body of literature suggesting ruxolitinib as a promising treatment option in SR-cGVHD.

## Key Points

**Question:**

Is ruxolitinib an option for patients with steroid-refractory chronic graft-vs-host disease, and what characteristics are associated with treatment response?

**Findings:**

In this case series of 41 patients with steroid-refractory chronic graft-vs-host disease who were treated with ruxolitinib, heavily pretreated patients could achieve meaningful responses with a favorable safety profile. No lung involvement and haploidentical donors were associated with response to ruxolitinib.

**Meaning:**

In this study, monotherapy with ruxolitinib was associated with a meaningful response in patients with steroid-refractory chronic graft-vs-host disease, suggesting a possible therapeutic option for a serious disease with no currently accepted standard-of-care treatment.

## Teaser

This case series investigates the clinical response to ruxolitinib in patients with steroid-refractory chronic graft-vs-host disease after allogeneic hematopoietic stem cell transplantation and evaluates its safety profile during the treatment course.

## Introduction

Chronic graft-vs-host disease (cGVHD) is the leading cause of late morbidity and mortality as well as impaired quality of life after allogeneic hematopoietic stem cell transplantation (HSCT).^1^ Despite the use of standard prophylaxis, 35% to 70% of recipients develop cGVHD.^2^ Established first-line therapy for cGVHD still comprises corticosteroids and calcineurin inhibitors.^3^ Approximately half of patients with cGVHD are refractory to corticosteroid therapy. For various second-line therapies or interventions, the response rates range from 30% to 60%,^4,5^ and no consensus has been reached regarding the optimal salvage treatment for steroid-refractory (SR)–cGVHD. Therefore, it is essential to identify a promising therapeutic drug for the adequate therapy of SR-cGVHD.

There is preclinical evidence that the pharmacologic inhibition of Janus kinases 1/2 (JAK1/J2) prevents GVHD by blocking interferon-γ receptor and interleukin 6 receptor signaling. JAK1/2 inhibition does not impair donor T-cell alloreactivity. It preserves the beneficial graft vs leukemia effect in vivo, suggesting a potential role for the selective JAK1/2 inhibitor ruxolitinib in the prevention and treatment of GVHD.^6,7^

A multicenter retrospective study in centers throughout Europe and the United States first established the feasibility of ruxolitinib for SR–acute GVHD (aGVHD) treatment.^8^ Thereafter, the Ruxolitinib for the Treatment of Steroid-Refractory Acute GVHD (REACH-1) study^9^ investigated ruxolitinib in treating SR-aGVHD and showed that the overall response rate (ORR) on day 28 was 54.9%. This was the first time such a study was conducted prospectively, and it shed light on later randomized clinical trials.^9^ Subsequently, a prospective study showed that combining ruxolitinib with another agent could achieve higher ORRs.^10^

At present, there is no standard of care for treating SR-cGVHD because of a lack of available and substantial data from prospective studies. The efficacy of ruxolitinib vs current best available therapy in SR-cGVHD is being evaluated in an ongoing phase 3 trial (REACH-3; NCT03112603).

Based on the limited preclinical and clinical outcomes of ruxolitinib in GVHD published to date, the present study investigated the clinical response to ruxolitinib and its safety profile in patients with SR-cGVHD after allogeneic HSCT. The study aimed to provide informative data for SR-cGVHD management and to measure the benefits and risks for different patient groups.

## Methods

This retrospective, single-center case series included 41 consecutive patients who were treated with ruxolitinib for SR-cGVHD after allogeneic HSCT between August 2017 and December 2019 at the First Affiliated Hospital of Zhejiang University School of Medicine. Informed consent was obtained from all recruited patients before ruxolitinib treatment and data collection. For safety and response evaluation, all data were collected from clinical history at the First Affiliated Hospital of Zhejiang University School of Medicine. The study was reviewed and approved by the ethics committee of the First Affiliated Hospital of Zhejiang University School of Medicine. This study followed the reporting guideline for case series.

All patients were aged 10 years or older and were successfully engrafted with full donor chimerism. Patients undergoing allogeneic HSCT, diagnosed with moderate or severe cGVHD, and refractory to steroid-based therapy were included. cGVHD was defined and graded following the National Institutes of Health (NIH) criteria or confirmed biopsy. SR was defined as cGVHD worsening on prednisone of at least 1 mg/kg/d for 1 to 2 weeks or sustained use of prednisone of a least 0.5 mg/kg/d (or 1 mg/kg every other day) for at least 4 weeks.^11^

Enrolled patients received ruxolitinib orally. Normally, a patient weighing 60 kg or less received a dose of 5 mg twice daily; patients weighing more than 60 kg received 10 mg twice a day. Patients with grade 3 cytopenia (according to National Cancer Institute–Common Terminology Criteria for Adverse Events [NCI-CTCAE] version 4.0) received 5 mg once a day. If patients presented worsening cGVHD according to NIH criteria within 4 weeks after administration, ruxolitinib would be withdrawn.

Response evaluation was conducted according to clinical status in the sixth month after the first ruxolitinib administration. The ORR included complete response (CR) and partial response (PR). CR was defined as the absence of all manifestations of cGVHD; PR was defined as an improvement in cGVHD compared with baseline clinical status and stage according to the NIH consensus and without any progression in any organs or sites. Other situations, including stable disease (SD), defined as no changes, and progressive disease (PD), defined as worsening in at least 1 site or organ, were categorized as treatment failure; discontinuation because of toxic effects from ruxolitinib was not included.

We collected and analyzed the following data: (1) adverse events, such as infections and cytopenia based on NCI-CTCAE version 4.0, were clinically relevant as grade 2 or higher; (2) time to response, defined as the initial use of ruxolitinib to initial response; (3) nonrelapsed mortality (NRM), which was defined as the initial treatment of ruxolitinib until death from any cause except underlying malignant neoplasm relapse or recurrence; (4) overall survival (OS), which was defined as the initial treatment of ruxolitinib until death from any cause; (5) cumulative incidence of cGVHD flare, which was defined as the initial use of ruxolitinib until cGVHD progression; and (6) the cumulative relapse of underlying malignant neoplasm, which was defined as the initial use of ruxolitinib until the first relapse. Patients who were lost to the last follow-up were censored.

### Statistical Analysis

Data were analyzed using SPSS statistical software version 22.0.01 (IBM Corp). A 2-tailed *P* < .05 was considered statistically significant. Univariate comparisons of parameters were performed using the χ^2^ test, Fisher exact test, and *t* test, as appropriate. Variables with *P* < .20 in univariate analysis were entered into the multivariate model. OS was estimated and plotted using the Kaplan-Meier method. The log-rank test was applied to compare Kaplan-Meier curves. The proportional-hazards method was used to estimate the cumulative incidence of relapse and NRM. Relapse and NRM were competing risks for each other. R statistical software version 3.4.3 (R Project for Statistical Computing) was used for the competing risk analysis.

## Results

### Patients

Between August 2017 and December 2019, 41 patients with SR-cGVHD, with a median (range) age of 31 (17-56) years and 14 (34.1%) women, were treated with ruxolitinib and included in this study. The demographic and baseline characteristics of the 41 participants are summarized in Table 1. Acute lymphoid leukemia (18 [43.9%]) and acute myeloid leukemia (17 [41.5%]) were the 2 most common diagnoses. All peripheral blood stem cells were obtained from related donors, including 9 patients (22.0%) receiving human leukocyte antigen (HLA)–matched grafts and 32 patients (78.0%) receiving HLA-haploidentical grafts. Only 1 patient (2.4%) underwent reduced-intensity conditioning, and antithymocyte globulin was administrated to the 32 patients (78.0%) receiving HLA-haploidentical transplantation (Table 1).

**Table 1.** Patient, Donor, and Transplant Characteristics

Characteristic	No. (%)
Patient age, median (range), y	31 (17-56)
Men	27 (65.9)
Male donor	20 (48.8)
Haploidentical donor	32 (78.0)
HLA-matched relative	9 (22.0)
Donor-recipient gender	
FF	9 (22.0)
MM	19 (46.3)
FM	10 (24.4)
MF	3 (7.3)
Stem cell source	
PBSC	41 (100)
Diagnosis	
ALL	18 (43.9)
AML	17 (41.5)
Other	6 (14.6)
Disease status at transplant	
CR	37 (90.2)
PR	2 (4.9)
Progressive	2 (4.8)
Conditioning regimen	
MAC	40 (97.6)
RIC	1 (2.4)
Use of ATG	
Yes	32 (78.0)
No	9 (22.0)
Prior acute graft-vs-host disease	
No	8 (19.5)
Grade I to II	21 (51.2)
Grade III to IV	12 (29.3)

Abbreviations: ALL, acute lymphoid leukemia; AML, acute myeloid leukemia; ATG, antithymoglobuline; CR, complete remission; FF, female to female; FM, female to male; HLA, human leukocyte antigen; MAC, myeloablative conditioning; MF, male to female; MM, male to male; PBSC, peripheral blood stem cell; PR, partial remission; RIC, reduced intensity conditioning.

A total of 32 patients (78.0%) developed prior aGVHD, with grade I (11 [34.4%]), grade II (10 [24.4%]), grade III (8 [25.0%]), and grade IV (4 [12.5%]). The median (range) time from HSCT to cGVHD diagnosis was 9.0 (3.3-27.2) months. Two patients (4.9%) showing overlap syndrome were enrolled in the study. At the time of enrollment, 27 patients (65.9%) had severe cGVHD, and 14 patients (34.1%) had moderate cGVHD. Organ involvement included skin (28 [68.3%]), mouth (29 [70.7%]), lungs (18 [43.9%]), liver (15 [36.6%]), eyes (14 [34.1%]), musculoskeleton (9 [22.0%]), kidney (3 [7.3%]), genitalia (3 [7.3%]), gastrointestinal tract (2 [4.9%]), eosinophilia (2 [4.9%]), nails (2 [4.9%]), and others (3 [7.3%]) (Table 2). More than half of patients (24 [58.5%]) had more than 2 sites or organs involved. No patient received posttransplant cyclophosphamide as GVHD prophylaxis.

**Table 2.** Characteristics of cGVHD

Characteristic	No. (%,)
Time from transplantation to cGVHD, median (range), mo	9.0 (3.3-27.2)
Overlap syndrome	2 (4.9)
Severe NIH score	27 (65.9)
Moderate NIH score	14 (34.1)
Organ affected	
Skin	28 (68.3)
Mouth	29 (70.7)
Lungs	18 (43.9)
Liver	15 (36.6)
Eyes	14 (34.1)
Joint and fascia	9 (22.0)
Genitalia	3 (7.3)
Kidney	3 (7.3)
Gastrointestinal tract	2 (4.9)
Nails	2 (4.9)
Involved sites	
1	6 (14.6)
2	11 (26.8)
3	13 (31.7)
4	5 (12.2)
>4	6 (14.6)
Previous second-line agents, median (range)	3 (1-6)
Previous agents	
Tacrolimus	36 (87.8)
Mycophenolate mofetil	26 (65.0)
Imatinib	15 (36.6)
CsA	15 (36.6)
Methotrexate	12 (29.3)
Mesenchymal stem cells	9 (22.0)
Infliximab	7 (17.1)
Others	10 (24.3)
Time from cGVHD to initial ruxolitinib treatment, median (range), mo	11.0 (0.6-71.9)

Abbreviations: CsA, cyclosporine A; cGVHD, chronic graft-vs-host disease; NIH, National Institutes of Health.

The median (range) number of second-line immunosuppressive agents before ruxolitinib was 3 (1-6). Tacrolimus (36 [87.8%]) and mycophenolate mofetil (26 [65.0%]) were the most commonly used agents. Ruxolitinib was started at a median (range) of 11.0 (0.6-71.9) months after the diagnosis of cGVHD (Table 2).

### Response, Outcomes, and Long-term Survival

**Figure 1.**
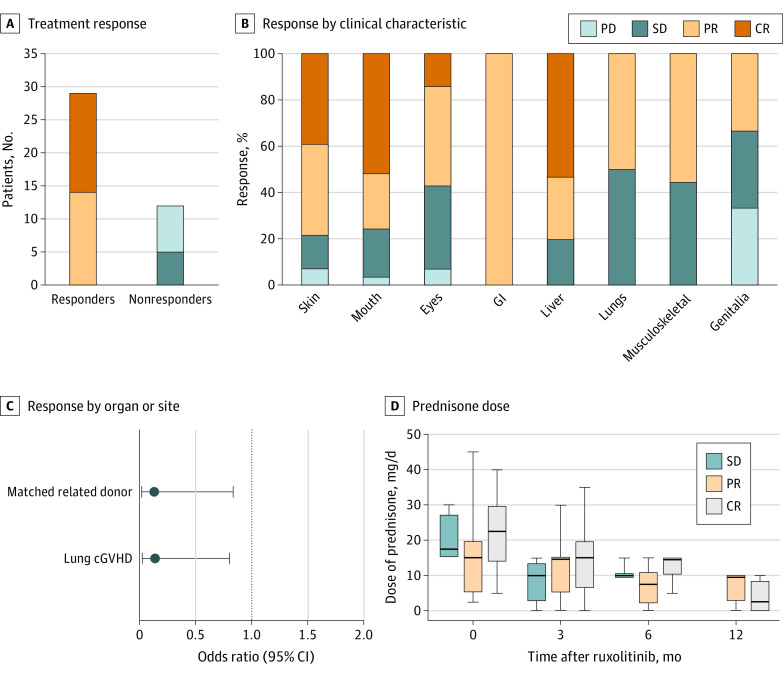
Treatment Response and Prednisone Dose Among 41 Patients with Steroid-Refractory Chronic Graft-vs-Host Disease (cGVHD) Median (range) treatment duration was 8.6 (1.1-24.9) months in responders. CR indicates complete remission; GI, gastrointestinal tract; PD, progressive disease; PR, partial remission; and SD, stable disease.

After a median (range) duration of 7.5 (1.0-24.9) months of ruxolitinib treatment, the ORR at 6 months was 70.7% (29 of 41 patients), including 15 patients (36.6%) with CR and 14 patients (34.1%) with PR. Among 12 patients (29.3%) with treatment failure, 5 (41.7%) had SD and 7 (58.3%) had PD (Figure 1A). The median (range) time to response was 2 (0.5-6.0) months. Ruxolitinib treatment was continued for a median (range) of 8.6 (1.1-24.9) months in those who responded. Patients with moderate cGVHD and those with severe cGVHD had similar ORRs (12 of 14 [85.7%] vs 17 of 27 [63.0%]; *P* = .17) and time to response (median [range], 2.3 [0.5-6.0] months vs 2.0 [1.0-6.0] months; *P* = .66). The number of involved organs was not associated with ORR or the time to response. There was no significant difference between patients who responded vs those who did not regarding previous lines of second-line agents (median [range], 4 [1-5] vs 3 [1-6]; *P* = .60) or time from cGVHD diagnosis to receiving ruxolitinib (median [range], 9.0 [0.6-71.9] months vs 14.1 [1.7-67.4] months; *P* = .89). Seven patients (17.1%) had ruxolitinib duration of less than 6 months because of death events (4 patients [57.1%]) and self-withdrawal due to favorable responses (3 patients [42.9%]). Compared with patients without lung cGVHD, the global ORR of those with lung involvement was relatively low (20 of 23 [87.0%] vs 9 of 18 [50.0%], *P* = .01). The ORR was 22 of 28 (78.6%) in the skin, 22 of 29 (75.9%) in the mouth, 8 of 14 (57.1%) in eyes, 2 of 2 (100%) in the gastrointestinal tract, 12 of 15 (80.0%) in the liver, 9 of 18 (50.0%) in the lungs, 5 of 9 (55.6%) in the musculoskeleton, and 1 of 3 (33.3%) in genitalia (Figure 1B). In this cohort, 18 patients (43.9%) discontinued ruxolitinib administration because of treatment failure (11 [61.1%]), relapse of underlying malignant neoplasm (6 [33.3%]), and lung infection (1 [5.6%]).

Variables with *P* < .20 in the univariate analysis were included in the later multivariate analysis. These were donor sex, recipient sex, donor type, history of donor lymphocyte infusion, cGVHD severity, lung involvement, and skin involvement. The logistic regression demonstrated that matched related donors (odds ratio [OR], 0.149; 95% CI, 0.022-0.981; *P* = .048) and lung cGVHD (OR, 0.112; 95% CI, 0.020-0.639; *P* = .01) were associated with treatment failure (Figure 1C). Although lacking statistical significance, the haploidentical HSCT group had higher ORR than the matched-related HSCT group (25 of 32 [71.4%] vs 4 of 9 [44.4%]; *P* = .09). There was no significant difference between the haploidentical group and the matched group in cGVHD baseline, including cGVHD severity (severe cGVHD: 19 [59.3%] vs 8 [88.9%]; *P* = .13), number of previous second-line drugs (median [range], 3 [1-6] vs 3 [2-5]; *P* = .28), duration of cGVHD course (median [range], 9.6 [0.6-71.9] months vs 16.4 [1.0-44.0] months; *P* = .90), or involved organs (skin: 24 [75.0%] vs 4 [44.4%]; *P* = .11; mouth: 22 [68.8%] vs 7 [77.8%]; *P* = .70; lung: 14 [43.8%] vs 4 [44.4%]; *P* > .99; liver: 11 [34.3%] vs 4 [44.4%]; *P* = .70; eyes: 9 [28.1%] vs 5 [55.6%]; *P* = .23; joint and fascia: 6 [18.8%] vs 3 [33.3%]; *P* = .38).

Furthermore, 26 of 29 patients who responded and 4 of 5 patients with SD (30 of 34 [88.2%]) received concomitant prednisone at a median (range) dose of 15 (2.5-45) mg/d without any dose alteration within 2 weeks before the first administration of ruxolitinib. At the last dose of ruxolitinib, prednisone reduction occurred in 27 patients (90.0%) at a median (range) dose of 10 (0-20) mg/d, with a median (range) reduction of 50.0% (33.3%-100%). Of these 27 patients, dose was decreased for 19 (63.3%) and discontinued for 8 (26.7%). The median (range) dose of concomitant prednisone at 3 months, 6 months, and 12 months was 15 (0-35) mg/d, 10 (0-15) mg/d, and 7.5 (0-10) mg/d, respectively (Figure 1D).

In 23 patients (56.1%) receiving concomitant second-line immunosuppressive agents, including 2 patients with SD and 21 who responded, dosage reduction and discontinuation were observed in 15 patients (65.2%) and 6 patients (26.1%), respectively. Only 2 of 14 patients (14.3%) with PR continued tacrolimus with the same dose for maintenance.

**Figure 2.**
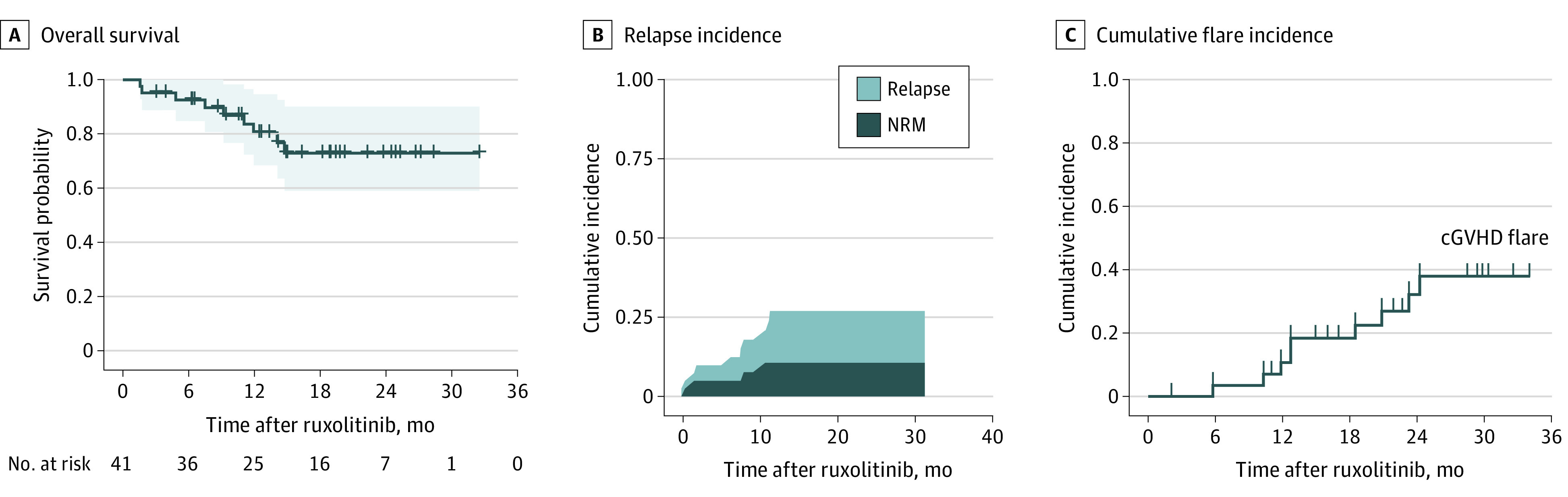
Overall Survival, Relapse Incidence, and Cumulative Flare Incidence During the Follow-up Period cGVHD indicates chronic graft-vs-host disease; NRM indicates nonrelapsed mortality.

**Figure 3.**
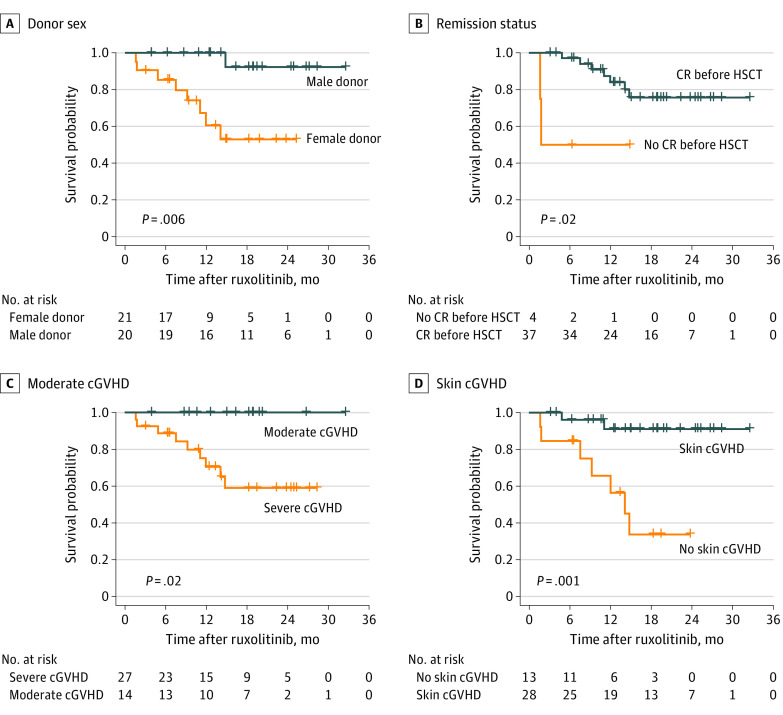
Survival Probabilities cGVHD indicates chronic graft-vs-host disease; CR indicates complete remission; and HSCT indicates hematopoietic stem cell transplantation.

The 6-month and 12-month OS rates for all treated patients were 87.8% (95% CI, 77.3%-98.3%) and 65.9% (95% CI, 50.7%-81.0%), respectively (Figure 2A). The median (range) follow-up was 14.9 (1.4-32.5) months. The univariate analysis showed that patients with a male donor (*P* = .006), complete remission before transplantation (*P* = .02), baseline moderate cGVHD (*P* = .02), and skin cGVHD (*P* = .001) might achieve prolonged survival (Figure 3). Grafts from a male donor were more likely to have skin cGVHD (18 of 20 [90.0%] vs 10 of 21 [47.6%]; *P* = .009), and most matched related donors were women (8 of 12 [66.7%] vs 1 of 20 [5.0%]; *P* = .02).

### Safety Profile

All adverse effects occurring after ruxolitinib administration were documented (eTable in the Supplement). The prophylaxis against infection, including sulfamethoxazole for pneumocystis pneumonia and micafungin or posaconazole for invasive fungal disease, was provided for all patients. Eleven patients (26.8%; 6 [54.5%] with response and 5 [45.5%] with no response) experienced lung infection during ruxolitinib treatment, and 3 patients (27.3%) died due to the infection. Patients with lung involvement were more likely to develop lung infection (8 of 18 [44.4%] vs 3 of 23 [13.0%]; *P* = .04).

Cytomegalovirus (CMV) detection before and after the use of ruxolitinib was performed for 34 patients (82.9%), of whom 5 patients (14.7%) underwent CMV DNAemia. Similarly, Epstein-Barr virus (EBV) data for 7 patients were unavailable, and 19 of 36 patients (52.7%) experienced EBV DNAemia after ruxolitinib administration. Two patients (4.9%) and 1 patient (2.4%) had carbapenem-resistant *Klebsiella pneumoniae* sepsis and hepatitis B (HBV) reaction, respectively.

Cytopenias were reported during ruxolitinib treatment in 6 patients (14.6%), of which 3 (50.0%) were of grades 3 to 4, including 1 grade 4 thrombocytopenia and 2 grade 3 leukocytopenia/thrombocytopenia. In 1 patient (16.7%), thrombocytopenia was related to the use of sulfamethoxazole.

Eight patients (19.5%) had manifestations of necrosis of the femoral head. Overall, the recurrence of underlying malignant neoplasm was found in 6 patients (14.6%). The cumulative incidence is shown in Figure 2B. cGVHD flare was observed in 9 of 29 patients (31.0%) with response due to drug discontinuation, presenting mild cGVHD manifestations (Figure 2C). These patients had cGVHD flare in their previously involved organs, including mouth, skin, musculoskeleton, and kidney. Four patients (44.4%) with cGVHD flare restarted ruxolitinib and regained initial responses. Overall, 9 patients died (22.0%) due to relapse of the primary malignancy (5 [55.6%]), pneumonia (3 [33.3%]; *Pneumocystis jiroveci* pneumonia, invasive fungal disease, and fungal/bacteria mixed infection), and acute liver failure caused by HBV reaction (1 [11.1%]).

The 1-year and 2-year NRM rates were 8.6% and 12.5%, respectively. The 1-year and 2-year cumulative incidences of relapse were 10.6% and 17.1%, respectively (Figure 2B).

## Discussion

The treatment strategies for patients with SR-cGVHD have changed. Multiple immunosuppressive agents provide clinicians with new approaches for treating patients with SR-cGVHD with few guidelines and a lack of consensus. Previous studies showed that the ORR of ruxolitinib in refractory cGVHD treatment was approximately 43.5% to 100%, and the CRR was from 3.5% to 13.0%, with a median time to best response ranging from 2 to 4 weeks.^8,12,13,14^ In the present study, the ORR was 70.7% (95% CI, 56.2%-85.3%), and the CRR was 36.6% (95% CI, 21.2%-52.0%). The median time to reach the best response was 2.0 months.

Ruxolitinib showed potential to resolve cGVHD. This study found that compared with patients with moderate cGVHD and less organ involvement, patients with severe and multiple involved organs had similar treatment outcome in terms of not only ORR but also the time to achieve response. Importantly, the treatment response showed no significant difference in patients with different previous lines of second-line agents and the time from cGVHD diagnosis to receiving ruxolitinib. The aforementioned scenario might suggest the use of ruxolitinib in patients with cGVHD regardless of cGVHD severity, the numbers of involved organs, the duration of cGVHD course, or the intensity of previous pharmacological therapies.

A primary aim of this study was to examine the factors associated with treatment response for ruxolitinib. The statistical results showed an association between lung cGVHD and treatment response, which was also reported in a previous study.^13^ The multivariate analysis indicated that among patients with cGVHD, lung involvement was associated with a higher risk of treatment failure.

The underlying mechanism of this association has not yet been elucidated. However, ensuing pulmonary fibrosis, symbolized with myofibroblast hyperplasia, is promoted by macrophages, B-cells, and complicated networks of other cells.^15^ A 3-phase model was developed for cGVHD in which the third phase is hypothesized to be due to the excessive accumulation of extracellular matrix, causing abnormal fibrosis.^16^

For example, interleukin 21 (IL-21) promotes the differentiation of B-cells into plasma cells via the JAK/signal transducer and activator of transcription 3 (STAT3) pathway, leading to antibody secretion and deposition.^17^ As an inhibitor of JAK-STAT signaling, ruxolitinib interferes with the activation and differentiation of T-cells and suppresses the activity of macrophages.^18^ However, the efficacy of ruxolitinib is not evident when it comes to the irreversible third phase, which is characterized by aberrant depositions and fibrosis. This explains the less effective results of ruxolitinib in lung cGVHD. In addition to treatment response, patients with lung cGVHD were more likely to develop pulmonary infection than patients with other types of cGVHD (8 of 18 [44.4%] vs 3 of 23 [13.0%]; *P* = .04). As such, patients without lung involvement were more likely to benefit from ruxolitinib. However, even the lungs could achieve a promising ORR of 50.0% in terms of difficulties in treating lung cGVHD. The ORR of other organs was similar, indicating that the pharmacokinetics of ruxolitinib could enable the distribution of the drug in target sites.

In this cohort, there was no significant difference between the haploidentical group and the matched group in cGVHD baseline, including cGVHD severity, number of previous second-line drugs, duration of cGVHD course, and involved organs. However, it is interesting to note that patients receiving haploidentical HSCT were observed to have higher ORR than those with matched related donors. Although JAK regulates the function of panoramic immune cells such as T-cells, B-cells, macrophages, and dendritic cells,^7,19,20,21^correlating with the etiology of cGVHD, the different treatment responses might underlie the heterogeneous pathogenesis of cGVHD between patients receiving stem cells from haploidentical and matched related donors. Currently, cGVHD is considered an entity, tantamount to an autoimmune disorder, with few comparisons in recipients receiving haploidentical and matched grafts. Thus, the potential disparities in the pathogenesis of cGVHD remain to be explored.

The cohort showed that male donors, CR before transplantation, skin cGVHD, and moderate cGVHD were associated with prolonged survival. This study found that patients receiving peripheral blood stem cells (PBSC) from male donors had more skin cGVHD events.

The estimated OS was compared between patients with and without a response. That the estimated OS plots were similar might ignore the fact that patients who responded had a higher quality of life, without or with fewer cGVHD events. A recent study demonstrated that the development of cGVHD was not associated with OS.^22^ In other words, survival time was comparatively fixed despite the resolution of cGVHD with the treatment of ruxolitinib. In this study, 23 patients had infections, including 11 with lung infection, 5 with CMV DNAemia, 19 with EBV DNAemia, 2 with sepsis, and 1 with HBV reaction. The latest REACH-1 study^9^ on aGVHD showed that infection events during ruxolitinib were approximately 80.3%, of which CMV events were the most common (19.7%). Zeiser et al^8^ found that the incidence of CMV events was higher in SR-aGVHD than in SR-cGVHD (33.3% vs 14.6%). Collectively, infection in patients with SR-cGVHD was tolerated, but 4 of 9 patients died of infection-related complications, highlighting the importance of antibacterial, antifungal, and antiviral prophylaxis.

Regarding cytopenia, the most common adverse effects related to ruxolitinib in the REACH-1 study were anemia (35.2%), thrombocytopenia (32.4%), and neutropenia (26.8%). The study by Zeiser et al^8^ showed that 5 patients with SR-aGVHD were more likely to develop global cytopenias and severe cytopenias compared with patients with cGVHD during the ruxolitinib course. The overall frequency of cytopenias in this study was only 14.6%, and grade 3 to 4 cytopenia occurred in 3 cases (7.3%), which was lower than the values reported in the study by Zeiser et al^8^ on the use of ruxolitinib in cGVHD and aGVHD.

### Limitations

This study has limitations. First, this single-center study lacked sufficient participants and death events to perform a multivariate analysis. For example, we found no statistical significance of OS between patients who did and did not respond. The limited data size dramatically interfered with the *P* value, which might lead to nonsignificant results, and patients from a single center might contribute to survival bias. It is important to emphasize that because of the study’s observational and retrospective nature, the results should be interpreted with caution. Second, some results failed to provide key insights. While we found patients with haploidentical donors might receive more benefit from ruxolitinib compared with matched related donors, the underlying mechanism for this phenomenon remains unclear. Third, although we highlight that ruxolitinib as a single agent could lower expenditure, we could not measure the actual costs of ruxolitinib regarding its dosage and course of treatment.

## Conclusions

This case series found that ruxolitinib in patients with SR-cGVHD had an ORR of 70.7% and CRR of 36.6%. Nearly all patients reduced the dose or discontinued the use of concomitant corticosteroids and other immunosuppressive drugs, minimizing their side effects and cost burden. Despite the limited sample size and retrospective nature, the results of this study indicated that patients with no lung involvement and haploidentical relatives as donors were more likely to benefit from ruxolitinib. Regarding the safety profile, the present study showed that infection events were the most severe adverse effect related to ruxolitinib, highlighting the significance of infection prophylaxis.

## Notes

Supplement. **eTable. **Safety Profile of Ruxolitinib
