# Supplementary material for: Evaluation of Ruxolitinib for Steroid-Refractory Chronic Graft-vs-Host Disease After Allogeneic Hematopoietic Stem Cell Transplantation
Source: JAMA Netw Open. 2021 Jan 27;4(1):e2034750. doi: 10.1001/jamanetworkopen.2020.34750 (PMC7841467; doi:10.1001/jamanetworkopen.2020.34750)
Supplement: Supplement. — eTable. Safety Profile of Ruxolitinib [file jamanetwopen-e2034750-s001.pdf]

Supplemental Online Content

Wu H, Shi J, Luo Y, et al. Evaluation of ruxolitinib for steroid-refractory chronic graft-vs-host disease after allogeneic hematopoietic stem cell transplantation. *JAMA Netw Open*. 2021;4(1):e2034750. doi:10.1001/jamanetworkopen.2020.34750

eTable. Safety Profile of Ruxolitinib

This supplemental material has been provided by the authors to give readers additional information about their work.

© 2021 Wu H. *JAMA Network Open.*

eTable. Safety Profile of Ruxolitinib

Adverse effects	Cases (%)
Infection	
Lung infection	11/41(26.8)
CMV reactivation	5/34(14.7)
EBV reactivation	19/36(52.7)
Sepsis	2/41(4.9)
HBV reaction	1/41(2.4)
Cytopenia	6/41(14.6)
Grade 1-2	3(7.3)
Grade 3-4	3 (7.3)
Malignancy relapse	6/41(14.6)
Necrosis of femoral head	8/41(19.5)

Abbreviations: CMV, Cytomegalovirus; EBV, Epstein-Barr virus; HBV, hepatitis B virus.

© 2021 Wu H. *JAMA Network Open.*
